# Advancing dental education with technology: The integration of smartphone applications in endodontics–A narrative review

**DOI:** 10.1111/iej.14219

**Published:** 2025-03-12

**Authors:** Seyed AmirHossein Ourang, Fatemeh Sohrabniya, Soroush Sadr, William A. Kahler, William Nguyen Ha

**Affiliations:** ^1^ Dentofacial Deformities Research Center, Research Institute of Dental Sciences Shahid Beheshti University of Medical Sciences Tehran Iran; ^2^ Dental Research Center Tehran University of Medical Sciences Tehran Iran; ^3^ Department of Restorative and Reconstructive Dentistry, The Sydney Dental School The University of Sydney Surry Hills New South Wales Australia

**Keywords:** dental education, endodontics, mobile app, smartphone applications

## Abstract

**Background:**

Smartphone applications are increasingly used in medical and dental education, offering flexible and interactive learning opportunities. In medicine, these tools enhance knowledge retention, clinical decision‐making, and patient care.

**Objective:**

This review aims to evaluate the role of smartphone applications in endodontic education, assessing their quality, functionality, perceived impact, usability, and impact on learning outcomes.

**Methods:**

A systematic search of the App Store, Google Play, PubMed, and Scopus was conducted to identify relevant applications. The quality assessment of the apps was performed through heuristic evaluation and the uMARS tool. The Inclusion criteria focused on apps designed for endodontic education and clinical practice.

**Results:**

From 350 records, 9 applications met the criteria. The heuristic rating for all apps was low to medium, except for Endolit, which demonstrated severe usability issues. Dental Endo Master received the best mean score in the uMARS rating. The selected applications offer diverse functionalities, including educational tools (EndoPrep, Endo Lit, Dental Endo Master, Adat Endodontic Cram Cards, Tooth SOS, Injured Tooth, AcciDent), diagnostic aids (Endo10), and case difficulty assessments (AAE Endo Case). Most apps were available on Android and iOS, with ratings ranging from 4.2/5 to 5/5.

**Discussion:**

This study highlights the growing role of smartphone applications in endodontics, particularly in education, clinical support, and patient guidance. While these apps offer interactive learning and diagnostic assistance, their quality varies due to differences in developer expertise, usability, and regulatory oversight. Some excel in engagement and functionality, while others require improvements in customization and accessibility. Ensuring evidence‐based development, standardized evaluations, and user‐centered design is essential for maximizing their impact on dental education and clinical practice.

**Conclusion:**

Smartphone applications have transformative potential in endodontic education, enabling flexible learning and improved clinical skills. However, challenges such as inconsistent quality and limited validation require attention.

**Registration:**

No formal registration was required for this narrative review.

## INTRODUCTION

In recent years, the digital revolution, propelled by advancements in internet and mobile technology, has paved the way for innovative educational tools that have significantly enhanced the learning experience for healthcare professionals (Hillenburg et al., 2006). Technology‐enhanced learning has become the cornerstone of medical and dental education, leveraging hardware like computers and mobile devices, and software such as mobile applications (Scott et al., 2017). With the widespread use of smartphones, mobile applications have emerged as influential tools in medical and dental education by providing users with access to evidence‐based information, educational materials, and practical tools at their fingertips (Chandran et al., 2012).

The integration of mobile applications in dentistry has revolutionized traditional learning methods by offering students and professionals convenient access to educational content anytime and anywhere (Sharples, 2013). Unlike conventional classroom‐based learning, mobile apps provide flexibility and adaptability to individual schedules, fostering a more engaging and immersive learning experience (Short et al., 2014). These apps can simulate real‐world scenarios and clinical procedures and allow students to practice and hone their skills in a controlled environment (Kratzke & Cox, 2012). Studies have shown that technology‐enhanced learning, including the use of mobile apps, leads to improved retention of knowledge, enhanced clinical skills, and greater learner satisfaction (Dror et al., 2011). Many dental education apps incorporate multimedia elements such as videos, animations, and interactive quizzes, promoting active engagement and catering to various learning styles (Khatoon et al., 2014).

In endodontics, mobile applications have become indispensable tools, enhancing clinical decision‐making and facilitating knowledge dissemination. Since the first simulation programs for endodontic instruction in 1987, the field has evolved significantly, now encompassing advanced artificial intelligence models and mobile apps (Ourang et al., 2024; Sandoval et al., 1987). A study in 2019 reported that 612 oral health‐related apps are available in the App Store, underscoring the growing interest in this technology (Fijačko et al., 2020). Apps designed for endodontists often include features such as case difficulty assessment (Shah et al., 2021), diagnostic tools (Mohan et al., 2018), and treatment planning support (Nalci et al., 2022), significantly improving patient outcomes.

This review aims to evaluate the role of smartphone apps in endodontic education and discuss their potential, limitations, and impact on learning outcomes. A narrative review of the available evidence will provide valuable insights into how these applications support clinical decision‐making, enhance knowledge dissemination, and promote proactive approaches to endodontic education and care.

## METHOD

This review was conducted following a systematic approach to identify smartphone applications relevant to endodontics. The primary goal of this review was to explore mobile apps designed to educate dental students and practitioners about endodontic problems and patient care.

### Search strategy and research question

We comprehensively searched endodontic‐related mobile applications across multiple platforms and databases. The search was performed in both the Apple App Store and Google Play Store, using the keywords “endodontics”, “dental trauma” and “root canal treatment.” Dental trauma‐based apps were also included, because they provide essential guidance for managing dental injuries, which are closely related to endodontic care. These apps help educate students and practitioners on the initial management of trauma cases that may later require endodontic intervention. To ensure the relevance and timeliness of the applications, the search was restricted to those released or updated until September 2024. In addition, an online literature search was performed using PubMed and Scopus, employing the terms “mobile application”, and “endodontic”, “root canal treatment”, or “dental trauma”. These databases were selected based on their extensive coverage of biomedical literature and relevance to dental education.

Based on the framework introduced by Cooke in 2012 (Cooke et al., 2012), the research questions were structured using the SPIDER criteria:

- Sample: Mobile apps.
- Phenomenon of Interest: Endodontics.
- Design: Survey and screening of apps.
- Evaluation: Education, functionalities, learning impact.
- Research Type: Qualitative

The primary question of this review was to explore mobile applications designed to educate dental students and practitioners about endodontic issues and patient care, while evaluating their quality, functionality, and perceived impact based on established guidelines and tools.

### Inclusion and exclusion criteria

The titles and descriptions of the applications were screened based on the following inclusion criteria:

- The application is in English.
- The application is specifically designed for the education of dental students or practitioners, with a focus on endodontic diagnosis, treatment planning, and patient care.

The application provides resources for understanding or managing endodontic cases. Applications that met the following criteria were excluded from the review:

- Applications focused on general oral hygiene, including tooth‐brushing monitoring or dental health tracking.
- Applications that offer dental practice management tools or general clinic management software.
- Educational games or other resources primarily designed for paediatric dental education.
- Non‐English applications were excluded to ensure accessibility to a broader clinical and academic audience.
- Review articles or summaries that did not introduce or evaluate specific mobile applications in endodontics.

The Inclusion criteria for studies in the literature review were:

- Type of studies: Studies evaluating mobile applications in dental education.
- Type of participants: Dental students and practitioners.
- Types of intervention: Use of mobile applications for endodontic education and training.
- Types of outcome measures: Knowledge retention, clinical decision‐making improvement, and user engagement.
- Electronic & other resources searches: Only PubMed and Scopus were selected due to their comprehensive indexing of peer‐reviewed articles in dentistry and mobile health technology.
- Selection of studies and data extraction: Two reviewers (F.S & S.S) screened the studies and extracted the data.
  All identified apps were evaluated based on their title, function, and relevance to endodontics by two reviewers (S.S and F.S). Selected applications were downloaded on either Android or iOS platforms for further content analysis. To evaluate features of apps such as downloads, price, developers, and top users, apps on the App Annie platform (appannie.com) were searched. App Annie is an app ranking, analytics, and market intelligence platform focused on apps.

### Quality assessment of the apps

The quality, functionality, and user perception of mobile applications related to endodontic education were assessed using the uMARS tool, which was applied by two independent reviewers (SAH.O and F.S). Both authors independently assessed all ratings and rankings. The uMARS tool consists of a 20‐item scale, which measures app objective quality across 16 items and subjective quality through 4 items. Additionally, it includes a subscale that evaluates perceived impact with six items. The objective quality of each app was assessed across four key domains: (A) Engagement—examining whether the app is interactive, enjoyable, and engaging; (B) Functionality—assessing the app's performance, ease of navigation, and reliability; (C) Aesthetics—evaluating the app's visual appeal, including background colours, font selection, and overall theme consistency; and (D) Information—analysing the sources of content and their reliability. Each of these domains (A, B, C, and D) is rated using a 5‐point scale, ranging from 1 (inadequate) to 5 (excellent). The mean app quality score was determined based on these ratings, while subjective quality and perceived impact scores were reported separately (Stoyanov et al., 2016).

The mobile app's usability was assessed by an app developer following the heuristic evaluation criteria (Khowaja & Al‐Thani, 2020). The evaluation considered three key factors—frequency, impact, and persistence—to determine the severity of usability issues. Nielsen's 10 general usability principles were applied to all apps, assessing aspects such as: (1) the match between the system and the real world, (2) visibility of system status, (3) adherence to standards and consistency, (4) user control and freedom, (5) minimalist and aesthetic design, (6) flexibility and efficiency of use, (7) user support in recognizing, diagnosing, and recovering from errors, (8) error prevention, (9) recognition over recall, and (10) help and documentation availability. Each app was evaluated based on heuristic principles and severity ratings. The severity of identified issues was categorized as high, medium, or low. High‐severity issues were considered critical, significantly affecting usability, functionality, or user experience, potentially hindering essential tasks or leading to data loss. Such issues required prompt attention and resolution to prevent substantial negative consequences. Medium‐severity issues, while affecting usability and user experience, were less critical than high‐severity concerns.

### Statistical analysis

For the uMARS evaluation, the average scores from both reviewers were computed for each application. Inter‐rater reliability was evaluated using Kendall's coefficient of concordance for the uMARS. For the heuristic evaluation, severity ratings were categorized as “Low” “Moderate” or “High”

## RESULTS

**FIGURE 1:**
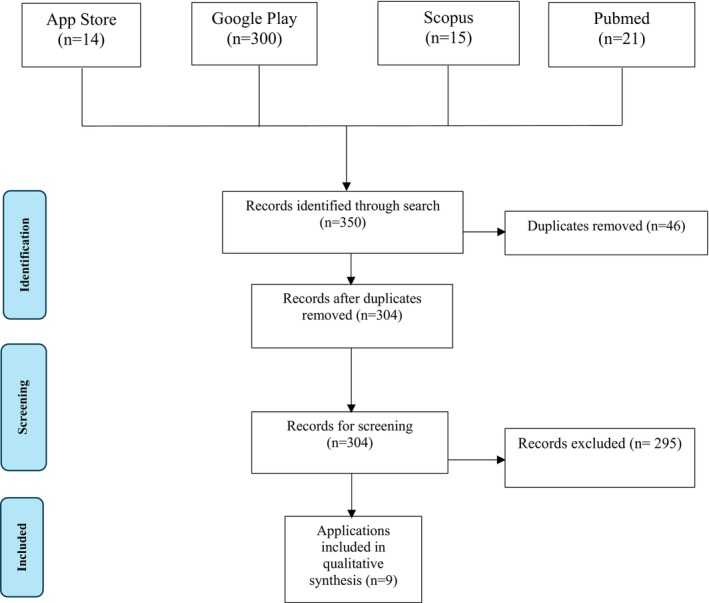
Prisma chart.

A search in both the App Store and Google Play using the selected keywords identified 314 smartphone applications. A literature review was also conducted, revealing 36 relevant articles (Figure 1). Among these, only one article introduced an application already identified through our search in the app stores.

During the screening stage, applications unrelated to endodontics were excluded based on their titles. Most excluded apps, particularly from Google Play, focused on oral hygiene tracking, dental health monitoring tools, and educational games designed for paediatric oral health. Duplicate applications between the App Store and Google Play were removed (*n* = 4). Non‐English applications were excluded.

Ultimately, 9 applications met all inclusion criteria and were incorporated into this review (Table 1).

**TABLE 1:** Applications related to endodontic education.

Application name	Developer	Platform	Rating	Country	Focus	Downloads	Price	Top users by country
Endo Prep	Dental Sciences Australia	Android, iOS	4.2/5	Australia	Education App for Dentists	APP Store: 5 K Google Play: 10–50 K	0.99 £	Google Play: Australia, Malaysia, Pakistan App Store: Australia, Malaysia, Saudi Arabia
Endo Lit	Adham Abdel Azim	Android, iOS	4.8/5	USA	Education App for Dentists	App Store: 5 K Google Play: 5–10 K	Free	Google Play: Denmark App Store: USA, Saudi Arabia, Egypt
AAE Endo Case	American Association of Endodontists	Android, iOS	4.6/5	USA	Case difficulty App	App Store: 5 K Google Play: 5–10 K	Free	Google Play: Egypt, Romania, Chile App Store: USA, Saudi Arabia, Australia
Dental Endo Master	Dental‐edutec	Android, iOS	N/A	N/A	Education App for Dentists	App Store: 5 K	One‐year full access: 39.99 £	App Store: Egypt, Russia, USA
Endo10	Crescendo Treinamentos Avancado LTDA	Android, iOS	5/5	Brazil	Diagnostic App	App Store: 5 K Google Play: 5–10 K	App Store:2.99 £ Google Play: Free	Google Play: Brazil, Mexico, Bolivia App Store: Brazil, Mexico, Peru
Adat Endodontic Cram Cards	Cram Cards	Android, iOS	N/A	USA	Education App for Dentists	App Store: 5 K Google Play: 1 K	App Store: 7.99 £ Google Play: 9.99£	Google Play: N/A App Store: Canada, Japan, Australia
ToothSOS	International Association of Dental Traumatology	Android, iOS	4.9	USA	Education App for Dentists and Patients	App Store: 5 K Google Play: 50–100 K	Free	Google Play: Turkey, Mexico, Spain App Store: Turkey, USA, Spain
Injured Tooth	Ignobillis Terrain	Android, IOS	N/A	India	Education App for Dentists	App Store: 5 K Google Play: 5 K	Free	Google Play: N/A App Store: India, Germany, Canada
AcciDent	Universitäres Zentrum für Zahnmedizin Basel	Android	N/A	Switzerland	Education App for Dentists	Google Play: 1 K	3.09 £	Google Play: Germany, Algeria, Venezuela

### Overview of selected apps

Out of the 9 selected apps, 7 apps are educational tools designed for dentists. These include EndoPrep, Endo Lit, Dental Endo Master, Adat Endodontic Cram Cards, Tooth SOS, Injured Tooth, and Accident. One app, AAE Endo Case, focuses on case difficulty assessment. Another app, Endo10, serves as a diagnostic tool. Finally, one app, ToothSOS, can be used by patients and dentists.

In terms of platform availability, 8 apps are accessible on both Android and iOS devices (EndoPrep, Endo Lit, AAE Endo Case, Endo10, ToothSOS, Injured Tooth, Dental EndoMaster and Adat Endodontics Cram Cards). One of the remaining apps is exclusive to the Android platform (Accident).

Regarding user ratings, 4 out of the 10 apps have published ratings. The average rating among these apps is approximately 4.72/5. Other apps do not have user ratings available. As for the country of origin, 4 apps originate from the USA (Endo Lit, AAE Endo Case, ToothSOS, and Adat Endodontic Cram Cards). One app, EndoPrep, is from Australia. Another app, Endo10, originates from Brazil. Injured Tooth and Accident are from India and Switzerland, respectively.

### Detailed review of the selected apps

The selected apps encompass a range of functionalities aimed at supporting endodontic practice and education:

EndoPrep is an educational app developed for dentists, dental students, and endodontists, and it is available on Android and iOS platforms. The app offers a range of free features, including measurement tools and calculators for length determination, radius, inclination, and curvature. It also provides guides for canal preparation, endodontic emergency management, and the use of loupes and microscopes, in addition to access to online webinars and articles. The premium EndoPrep Clinical version includes additional measurement and calculator features and a guide for detecting the MB2 canal. The app's innovative features include a D12 pericervical dentine tool that can calculate final preparation file size recommendations to preserve pericervical dentine based on factors like the first apical binding file, curvature, and root diameter. Users can also upload images to the app and have measurements calculated automatically.

Furthermore, after preparing the mesiobuccal, distobuccal, and palatal canals, users can upload the preparation shape, and the app will guide them to the likely location of the second mesiobuccal canal. EndoPrep has a 4.2/5 user rating (from 65 total ratings) on the Google Play Store, with 10 000–50 000 downloads. The primary user base is located in Australia, Malaysia, and Pakistan. On the App Store, the app has fewer than 5000 downloads and a 4.2/5 rating(from 5 total ratings), with users primarily from Australia, Malaysia, and Saudi Arabia. EndoPrep is a free download, with the premium EndoPrep Clinical version priced at £0.99.

EndoLit is a professional networking and educational application developed by Adham Abdel Azim and is available on Android and iOS platforms. The app is a comprehensive platform for endodontic education and professional networking among clinicians. Its key features include personalized user profiles, case‐sharing capabilities, professional networking tools with text messaging functions, and access to endodontic literature, lecture series, courses, and board preparation materials. The user interface facilitates social interaction through a forum‐style layout that enables case discussions, notifications for engagement (comments and likes), and easy access to educational resources. The educational value is enhanced by content oversight from moderators and a scientific advisory board comprising renowned clinicians and researchers in endodontics who curate featured articles and maintain the quality of forum discussions. The app has garnered positive user feedback with a high rating of 4.8/5 (from 315 total ratings) and has achieved moderate download numbers (less than 5000 on the App Store and 5000–10 000 on Google Play). Available as a free download, it has found particular popularity among users in Denmark through Google Play while maintaining a presence in the USA, Saudi Arabia, and Egypt through the App Store.

The AAE Endo Case is a specialized app developed by the American Association of Endodontists (AAE), available on Android and iOS platforms. The app is designed to assist practitioners in assessing the difficulty level of endodontic cases using the AAE's standardized case difficulty assessment form. Its key features include a comprehensive scoring system for case evaluation, an abridged version for quick assessments of common cases, and the ability to generate patient referral forms. The user interface presents criteria‐based selection options where users can match patient conditions to predetermined parameters, though it relies primarily on text‐based interactions. The app has received positive user feedback with a 4.6/5 rating (from 9 total ratings) and has achieved moderate download numbers (less than 5000 on the App Store and 5000–10 000 on Google Play). Available as a free download, it has gained particular traction among users in Egypt, Romania, and Chile on Google Play while maintaining a user base in the USA, Saudi Arabia, and Australia through the App Store.

Dental Endo Master is an educational app developed by Dental‐edutec, and it is available on iOS and Android. It presents a distinctive approach to endodontic education through its interactive 3D learning environment. The app simulates clinical scenarios by presenting a 3D tooth model within the oral cavity that users can freely manipulate, accompanied by corresponding radiographic images and a secondary view revealing internal tooth structures. The learning process follows a structured clinical workflow: users begin by planning and executing their initial access entry, progress to predicting canal locations, and finally design the definitive access cavity. What sets this app apart is its analytical feedback system that evaluates performance at each step, providing detailed scoring and constructive feedback to help users improve their technique. This systematic approach helps transform theoretical knowledge into practical skills through hands‐on virtual practice. Step‐by‐step guides and comprehensive tutorials complement the 3D features, ensuring users understand each stage of the procedure. The educational experience is enhanced through gamification elements, including achievement systems, challenge events, and rankings, encouraging continued practice and skill development. While basic features are free, full access requires a one‐year subscription at £39.99. The app has garnered fewer than 5000 downloads on the App Store, with users primarily based in Egypt, Russia, and the United States, though no ratings are currently available.

Endo10 is a diagnostic app developed by Crescendo Treinamentos Avancado, available on Android and iOS platforms. The app is designed to assist dental practitioners in diagnosing pulpal and periapical conditions by systematically evaluating patients' signs, symptoms, and radiographic data. The app's core functionality is centred around a structured set of 9 questions that cover essential diagnostic areas, such as the presence and nature of pain, pulp vitality, and periapical test findings. Endo10 provides a suggested final diagnosis to support clinical decision‐making by guiding users through this comprehensive assessment. To accommodate a wide range of users, the app is available in three languages: Portuguese, Spanish, and English. Endo10 has received a 5/5 user rating on the App Store, with fewer than 5000 downloads. On the Google Play Store, it has achieved 5000–10 000 downloads and maintains a 5/5 rating (from 2 total ratings). The primary user base for Endo10 is located in Brazil, Mexico, and Bolivia on the Google Play Store, while on the App Store, the app's users are concentrated in Brazil, Mexico, and Peru. The app is free on the Google Play Store, while the iOS version is priced at £2.99.

ADAT Endodontic Cram Cards is an educational app that supports dental students preparing for advanced programmes and dental school exams. It is available on iOS and Android. The app features digital flashcards with images, text, and slides to memorise key endodontic concepts efficiently. The app aims to enhance students' understanding of endodontic topics within a manageable timeframe by offering an interactive and structured learning approach. On the Google Play Store, it has fewer than 1000 downloads; on the App Store, it has recorded less than 5000 downloads. The iOS version is priced at £7.99, and the Android version is priced at £9.99. The app's primary user base is Canada, Japan, and Australia.

ToothSOS is a diagnostic app developed by the International Association of Dental Traumatology. The app is designed as a resource for dentists and patients, guiding them in identifying and managing different types of dental injuries and trauma. The app uses plain language and visual aids to help users assess dental emergencies. For example, it describes an avulsed tooth as “knocked out” and an intruded tooth as “pushed in.” ToothSOS offers step‐by‐step instructions for various trauma scenarios, advising users on preserving injured teeth. The app also includes a referral system to connect users with qualified dental trauma specialists.

Additionally, ToothSOS incorporates the clinical guidelines established by the International Association of Dental Traumatology, providing dentists with evidence‐based recommendations for managing dental trauma cases. The app is available on Android and iOS platforms with a 4.9/5 rating(from 125 total ratings). On the Google Play Store, it has achieved 50 000–100 000 downloads, while the App Store version has fewer than 5000 downloads. ToothSOS is offered as a free download. The primary user base for the Android version is located in Turkey, Mexico, and Spain, while the App Store version is most popular in Turkey, the USA, and Spain.

The Injured Tooth app is a comprehensive dental trauma management tool developed by Ignobillis Terrain, and it is available on both iOS and Android devices. The app is designed to assist dental professionals in diagnosing and documenting various dental injuries, including those affecting primary, permanent, and multiple teeth. Key features of the Injured Tooth app include a built‐in alert system for scheduling and tracking patient follow‐ups, as well as the ability to document cases through clinical notes and photographs. The app also provides direct access to the guidelines established by the International Association of Dental Traumatology (IADT), allowing clinicians to reference the latest evidence‐based recommendations for managing dental trauma. Additionally, the app incorporates an interactive quiz module that reinforces knowledge through case‐based scenarios. The Injured Tooth app is available on both Android and iOS platforms. On the Google Play Store, it has recorded fewer than 5000 downloads, while the App Store version has also achieved fewer than 5000 downloads. The app is offered as a free download. The primary user base for the Android version is currently unknown, while the App Store version is most popular among users in India, Germany, and Canada.

The AcciDent app, available on Android platforms, is a specialized tool developed by the Universitäres Zentrum für Zahnmedizin Basel to assist dental professionals in managing traumatic dental injuries. The app provides comprehensive information on a wide range of clinical scenarios related to dental trauma, from minor enamel cracks to more severe crown‐root fractures. Users can access detailed guidance on managing various dental injuries, including crown fractures with pulp involvement. The app features a bilingual interface that is available in English and German. On the Google Play Store, the AcciDent app has achieved fewer than 1000 downloads, with the primary user base located in Germany, Algeria, and Venezuela. The app is priced at £3.09 on the Google Play Store.

### Quality assessments using uMARS and heuristic evaluation

App measurements based on the uMARS scale were completed by two independent reviewers. The average scores for engagement, functionality, aesthetics, and information were calculated by dividing the sum of scores by the number of corresponding features, ensuring all scores were standardized on a scale out of 5. The scores from both reviewers were compiled, and the average of their ratings was determined for each measure, which was then entered into a consolidated table. Subsequently, the overall average score and subscale scores were computed based on these averaged values. Kendall's coefficient of concordance showed a good agreement between the two reviewers (0.79).

The evaluation of dental mobile applications using the uMARS framework revealed notable variations across different criteria (Table 2). Among the assessed apps, Dental Endo Master achieved the highest mean overall score (4.0/5), excelling particularly in engagement (4.2/5) and aesthetics (4.7/5), highlighting its interactive and visually appealing interface. However, apps like AcciDent (2.8) and Tooth SOS (2.9) received lower scores due to lower engagement and aesthetics ratings. Functionality ratings varied, with EndoPrep and EndoCase leading in ease of navigation and gestural design, while Dental Endo Master and ADAT had the highest credibility scores for information quality. Subjective quality assessments indicated that Endo master and EndoPrep were most likely to be recommended, while Tooth SOS and AcciDent had lower recommendation scores. In terms of perceived impact, EndoPrep demonstrated the strongest influence on awareness (4.5/5) and knowledge acquisition (4.0/5), whereas EndoLit and EndoCase scored moderately across these domains.

**TABLE 2:** Average scores for app quality and subjective quality ratings, along with perceived impact scores of the app, as measured by the uMARS Scale.

Section		Category	Endo prep	EndoLit	Endo case	Dental Endo master	Endo 10	Injured tooth	Tooth SOS	AcciDent	ADAT cram cards
App quality ratings	Engagement	Entertainment	4.5	4	3	4.5	3	3	2.5	2.5	3.5
		Interest	4	3.5	3	4	4	3	4	3	4
		Customization	1.5	3	1.5	3.5	3	3	1.5	2	3
		Interactivity	4	3	3.5	4.5	3.5	2.5	1.5	2	3.5
		Target Audience	4	4	4.5	4.5	4.5	4	3.5	4.5	3.5
		Mean Score(out of 5)	3.6	3.5	3.1	4.2	3.6	3.1	2.6	2.8	3.5
	Functionality	Performance	4.5	4	4.5	3.5	3.5	3	3.5	3	3.5
		Ease of Use	3.5	3	4	3	3.5	2.5	2.5	3.5	4
		Navigation	4.5	3	4.5	4.5	3	3	3	2.5	3.5
		Gestural Design	3.5	4	3.5	4	3.5	3	3.5	3	2.5
		Mean Score(out of 5)	4	3.5	4.1	3.4	3.4	2.9	3.1	3	3.4
	Aesthetics	Layout	3	3.5	4	4.5	3.5	2.5	2.5	2	2
		Graphics	2	2.5	3	5.5	3	2.5	2	2.5	2.5
		Visual Appeal	2.5	2.5	3	4	3	2.5	2.5	2.5	2.5
		Overall	2.5	2.8	3.3	4.7	3.1	2.5	2.3	2.3	2.3
	Information	Quality	4.5	3.5	4	3.5	3.5	4.5	4	3	3.5
		Quantity	4	3.5	3	3	4	4.5	3.5	4	4.5
		Visuals	3.5	3.5	4	4.5	4	3	3	3	3.5
		Credibility	4	4.5	5	4.5	4	4.5	4	2.5	5
		Mean Score(out of 5)	4	3.7	4	3.9	3.9	4.1	3.6	3.1	4.1
	Mean Overall Score		3.5	3.4	3.6	4	3.5	3.1	2.9	2.8	3.3
Subjective quality		Recommendation	3.5	3	3	4	3	3	2.5	2.5	3
		Frequency of Use	2.5	2.5	2.5	2.5	3	2	2	2	2
		Willingness to Pay	3	2	2	3	3.5	2	2.5	2.5	3.5
		Overall Rating	4	3	3.5	4	3.5	2.5	2.5	2.5	3
Perceived Impact		Awareness	4.5	3.5	4	3	4.5	2.5	3	2.5	3.5
		Knowledge	4	3.5	3.5	3	3	3	2.5	2.5	3.5
		Attitudes	3.5	3	3	3.5	3.5	2.5	3	1.5	3
		Motivation	3.5	2.5	3	3	3	3	2	3	3
		Help‐Seeking	2.5	3.5	3.5	3	3	2.5	3.5	3	3.5
		Behaviour Change	3.5	3	2.5	3	3.5	3	3.5	2	3

An experienced app developer conducted a heuristic evaluation of all the included apps. Most apps demonstrated low to moderate (1–4 features) heuristic ratings, except for EndoPrep, which had the highest number of heuristic features (12) but was rated at a medium severity level. EndoLit exhibited severe usability issues despite having nine heuristic features, indicating critical areas needing refinement. The severity of each app, along with Nielsen's ratings, is detailed in Table 3. A comprehensive heuristic evaluation report of all the included apps is provided in Data S1.

**TABLE 3:** Heuristic evaluations for the mobile apps included in the study.

App	No. of heuristic features	Nielsen's severity rating
EndoPrep	12	Medium
EndoLit	9	Severe
EndoCase	3	Low
Dental EndoMaster	1	Low
Endo 10	2	Medium
Injured Tooth	4	Medium
Tooth SOS	4	Medium
AcciDent	4	Low
ADAT Endodontic Cram Cards	4	Medium

## DISCUSSION

This study provides a comprehensive overview of the currently available smartphone applications in endodontics. Apps were systematically categorized based on their platform (Android, iOS, or both), target users (dentists, clinicians, patients, or both), number of downloads, and pricing structure (Table 1). Furthermore, apps' quality, functionality, user perception and usability were evaluated using uMARS and heuristic rating tools. The selected apps showcase a range of functionalities, platforms, and user interfaces designed to meet the needs of endodontists, dental students, and, in some cases, patients. Notably, applications such as Endo Prep, Endo Lit, AAE Endo Case, and Endo10 exemplify educational tools focused on clinician training. Apps, like ToothSOS, extend educational resources to patients, enhancing their understanding and management of dental trauma. The emphasis on educational apps for clinicians can be attributed to the intricate nature of endodontic procedures and the continuous need for professional development in this speciality. Smartphone apps offer a convenient and accessible platform for clinicians to stay abreast of the latest advancements, guidelines, and best practices (Pascadopoli et al., 2023). Mobile devices' portability and ease of use make them ideal for busy professionals who need quick access to information during clinical practice or patient consultations.

The focus on clinician education reflects the demand for interactive and multimedia learning resources that enhance traditional educational methods. Apps can provide interactive 3D models, virtual simulations, and case studies that are not possible with textbooks alone. Interactive learning tools, such as mobile applications and web‐based platforms, have shown promising results in enhancing knowledge retention, decision‐making skills, and practical outcomes in medical education. A randomized trial investigating the effectiveness of a smartphone application for public health service physicians in paediatric oral health care demonstrated significant improvements in knowledge, attitude, and practice post‐intervention. Although the smartphone‐based approach was not statistically superior to traditional continuing medical education (CME) methods, it proved to be an effective learning tool (Bonabi et al., 2019). Similarly, the integration of mobile Internet devices (MIDs) and proprietary applications in medical education has facilitated better information management, communication, and time management among caregivers (Mohapatra et al., 2015).

The quality of the apps varies significantly across the spectrum. One reason for this variation is the background and expertise of the app developers. Apps developed by endodontists and professionals (Endoprep, AAE endo case, Endolit, AcciDent and Tooth SOS) within the field tend to offer higher‐quality content that is more relevant and tailored to the needs of clinicians. These developers have firsthand experience with the challenges faced in endodontic practice and can design app features that address specific educational gaps or clinical decision‐making processes.

However, other factors contribute to the variation in app quality beyond the developers' professional backgrounds. The resources available for app development, such as funding, access to technological expertise, and collaboration with educational institutions, play a crucial role. Apps backed by professional organizations or universities may have more comprehensive content, better user interfaces, and more reliable information due to rigorous peer review and testing processes. Regular maintenance and periodic updates are essential to ensure compatibility with new technologies and maintain app functionality. For instance, the Injured Tooth app's performance significantly deteriorated on modern devices due to outdated programming.

The lack of standardized guidelines or regulatory oversight for medical and dental apps can lead to inconsistencies in content accuracy, usability, and overall effectiveness. Without clear standards, developers may produce apps that vary widely in quality, potentially impacting the educational value and reliability of the information provided. Another significant challenge in assessing the quality and user satisfaction of these apps is the scarcity of user reviews, comments, and ratings. Many apps lack sufficient user feedback, making it difficult to evaluate their effectiveness from the users’ perspective. This absence of feedback could be due to low download numbers, limited user engagement, or a reluctance among users to provide reviews. User ratings and comments are essential for identifying the strengths and weaknesses of the apps, guiding future improvements, and helping potential users make informed choices.

The heuristic evaluation provided further insights into the usability and interface design of the apps. EndoPrep demonstrated the highest number of heuristic features (12) with a medium severity rating, suggesting some usability concerns that require refinement but do not severely hinder user experience. EndoLit, with 9 heuristic features, received a severe severity rating, indicating significant usability challenges that may affect navigation and user engagement. Several apps, including EndoCase, Dental Endo Master, and AcciDent, had lower heuristic scores but low severity ratings, suggesting fewer critical usability issues. The medium severity ratings observed in apps like Tooth SOS, Injured Tooth, and ADAT Endodontic Cram Cards indicate moderate usability concerns that could benefit from design improvements, such as enhanced menu structures, improved consistency in design elements, and refined navigation pathways. These findings highlight the importance of optimizing interface intuitiveness, consistency, and accessibility to improve the overall user experience of endodontic mobile applications.

The uMARS evaluation of the included dental mobile applications highlights strengths and areas needing improvement. Engagement scores varied significantly across the apps, indicating that while some incorporated interactive and user‐centered design elements, others lacked essential features to maintain user interest. Dental Endo Master scored the highest in engagement due to its well‐structured interactivity and target audience focus, while Tooth SOS and AcciDent received lower engagement scores, likely due to limited customisation and user involvement. These findings emphasize the importance of enhancing interactivity, personalisation options, and entertainment value to improve user experience. Functionality ratings also showed variation, with EndoPrep and EndoCase leading in ease of navigation and intuitive design, suggesting that well‐structured menus and smooth transitions contribute to better usability. On the other hand, lower functionality ratings in some apps highlight the need for improved navigation flows and optimised performance to enhance usability across different user groups, including clinicians and patients.

Similar to our study, Walia et al. conducted a heuristic evaluation and analysis of apps related to dental trauma. They used uMARS (User Mobile Application Rating Scale), heuristic evaluation, and the Coventry, Aberdeen, and London–Revised (CALO‐RE) scale. The Tooth SOS and Injured Tooth apps demonstrated the best performance in both the uMARS and heuristic evaluations (Walia et al., 2024).

In another study, Roy et al. in 2022 conducted an availability and content analysis of endodontic apps using the Mobile Application Rating Scale (MARS). This scale assesses aspects such as engagement, functionality, aesthetics, information quality, and overall subjective quality based on 23 questions. The analysis revealed that the AAE Endo Case app, followed closely by the Endo 10 app, achieved the highest overall scores among endodontic applications. These findings highlight the potential of well‐designed smartphone apps to contribute positively to endodontic education and clinical practice (Roy et al., 2022). Although the results of quality assessments for these apps vary across studies, such differences are logical given the subjective nature of the ratings and the fact that app updates can lead to improved performance in subsequent evaluations.

A critical aspect of smartphone applications in healthcare is evaluating their utility, safety, and efficacy through clinical scenarios (Bender et al., 2013). Among the reviewed applications, only two applications, Endo 10 and Injured Tooth, have undergone such evaluations. Mohan et al. assessed the diagnostic accuracy of the Injured Tooth app in identifying traumatic dental injuries (Mohan et al., 2018). Their study found that diagnoses made by the app closely aligned with those made by experienced faculty members. These findings suggest that the Injured Tooth app can be a reliable tool for educating dental students and residents and supporting practitioners in managing dental trauma cases. Similarly, Abuabara et al. evaluated the Endo 10 app as a diagnostic tool in endodontics, focusing on its usability, usefulness, and reliability. They assessed the app's ease of use and technology acceptance through usability and usefulness tests. Reliability was gauged by comparing diagnostic outcomes provided by the app to those of experienced endodontists. The Endo 10 app demonstrated acceptable usability, usefulness, and reliability levels, showing promising results across all evaluation criteria (Abuabara et al., 2023).

Smartphone apps offer valuable support in dental education and case work‐up as practical supplements to traditional learning and clinical practice. They can effectively bridge the gap between theoretical knowledge and clinical practice in endodontics. Educational apps like AAE Endo Case and Dental Endo Master allow students and clinicians to explore endodontic concepts through interactive case assessments, 3D simulations, and detailed procedural guides. For instance, Dental Endo Master provides 3D models and guided simulations that enable users to practice endodontic techniques in a virtual environment, which is particularly beneficial for students preparing for clinical scenarios. AAE Endo Case offers structured case difficulty assessments, helping users systematically evaluate case complexities—skills crucial for treatment planning and patient management. Trauma‐focused apps such as Accident, ToothSOS, and Injured Tooth also serve educational and practical roles by guiding users through diagnosing and managing dental trauma. These apps provide step‐by‐step instructions and visual aids that benefit dental students and practitioners, enhancing preparedness for trauma cases.

Prognostic calculators within endodontic apps could be important in determining case eligibility for government or insurance support. By evaluating factors such as case complexity, anatomical difficulties, and patient‐specific variables, these tools can offer standardized metrics to aid in eligibility assessments for funding or insurance claims (Raber et al., 2019). For instance, AAE Endo Case and EndoPrep could be expanded to include prognostic calculators that generate data‐driven insights, supporting transparent and consistent coverage decisions. These features could improve access to necessary treatments, particularly for complex cases requiring significant resources, while fostering fairness and clarity in government and insurance support determinations.

In light of our findings, we recommend that developers of dental mobile applications adopt user‐centric approaches, employing tools such as uMARS during the development phase to evaluate prototypes and identify potential usability issues. Conducting usability testing prior to app release is crucial for enhancing user satisfaction and optimizing functionality. In addition, aligning development practices with guidelines like the mHealth evidence reporting and assessment (mERA) checklist is essential to ensure quality and consistency (Agarwal et al., 2016). Future studies should explore how intuitive and user‐friendly these applications are for both students and educators, and assess their impact on enhancing theoretical knowledge, practical skills, and case‐based learning in endodontics. Despite the potential of these apps, there are limitations such as heterogeneity in study designs and sample sizes and differing study durations, which complicate generalisability.

## CONCLUSION

This review underscores the increasing integration of smartphone applications in endodontic education and practice. The identified apps offer a spectrum of functionalities—including educational tools, diagnostic aids, case difficulty assessments, and addressing the diverse needs of dental professionals and students. While these applications provide valuable resources that enhance learning and clinical decision‐making, significant variability exists in their Functionality, usability, and perceived impact.

Overall, smartphone applications hold significant promise for innovation in endodontics, offering accessible, interactive, and up‐to‐date resources that complement traditional educational methods. These apps can become integral to endodontic education and practice by addressing current limitations, ultimately improving patient care and outcomes.

## AUTHOR CONTRIBUTIONS

**Seyed AmirHossein Ourang**: Conceptualization, Methodology, Data curation, Writing–original draft, Writing–review & editing. **Fatemeh Sohrabniya**: Methodology, Data curation, Writing–original draft, Writing–review & editing. **Soroush Sadr**: Conceptualization, Writing–review & editing. **William A. Kahler**: Conceptualization, Methodology, Data curation, Writing–review & editing. **William Nguyen Ha**: Conceptualization, Methodology, Data curation, Writing–review & editing.

## CONFLICT OF INTEREST STATEMENT

The authors confirm they have no conflict of interest.

## ETHICS STATEMENT

This study is a narrative review and does not require ethics approval.

## Supporting information


Data S1.

## Data Availability

Data sharing is not applicable.
