# Supplementary material for: Advancing dental education with technology: The integration of smartphone applications in endodontics–A narrative review
Source: Int Endod J. 2025 Mar 12;59(6):986–98. doi: 10.1111/iej.14219 (PMC13158530; doi:10.1111/iej.14219)
Supplement: Supplementary file 1 — Data S1. [file IEJ-59-986-s001.docx]

***Endoprep***

**Landing Page:**
The "EndoPrep Clinical" button, which facilitates access to the premium version, is currently displayed alongside functional features such as calculators and guides. To enhance user clarity and avoid potential confusion, it is recommended to relocate this feature to a side menu or a dedicated section. (Severity: 2)

**Duplicate Feature:**
The "D12 Pericervical Dentine" option is currently duplicated, appearing both on the landing page and within the radiographic measurement menu. Consolidating its placement into a single, logical location would improve navigation efficiency and reduce redundancy.. (Severity: 3)

**Excessive Clicks:**
Several features currently necessitate excessive clicks to access, which may hinder user efficiency. Adhering to the 3-click rule—ensuring that users can reach any feature within three clicks—would significantly enhance the user experience by minimizing unnecessary navigation steps. (Severity: 3)

**Interactive Button Visibility:**
The "Yes, begin preparation guide" button within the "Root Canal Preparation Guide" menu should be highlighted using a distinct color to enhance its visibility and emphasize its interactive nature, thereby improving user engagement and clarity. (Severity: 1)

**Help Button Placement:**
Relocating the help option to an interactive button positioned adjacent to each tool would enhance usability by minimizing confusion and reducing the number of clicks needed to access assistance, thereby streamlining the user experience. (Severity: 3)

**Interactive Input Design:**
Replacing the dropdown Yes/No options with interactive checkboxes (empty or filled) would improve the interface's intuitiveness, making it more user-friendly and visually straightforward for navigation. (Severity: 2)

**Heading Consistency:**
Inconsistencies between button text and page headings, such as the "Ideal Taper Size" heading versus the "D12 Pericervical Dentine" button, may lead to user confusion. To enhance clarity and usability, it is essential to maintain consistent labeling across all sections of the app.(Severity: 2)

**Study Guides Content:**
While the online study guides provide multiple resource links, the "Practice Essay Questions" section is currently brief, incomplete, and lacks relevant links. Expanding and enhancing this section with comprehensive content and additional resources would significantly increase its value and utility for users. (Severity: 1)

**Font and Graphics:**
The current font size is insufficiently small, and the app's graphics lack visual appeal. Incorporating vibrant colors and engaging visuals would enhance usability, create a more attractive interface, and ultimately improve user satisfaction. (General Observation)

**Content Organization:**
Separating the education section from taper, length, and outcome features, or offering different payment tiers for these sections, could cater to the varied needs of endodontic courses. (General Observation)

**Complex Cases Handling:**
It is unclear how the app manages complex cases. Adding endorsements or recommendations from prominent figures in the field would increase trust and usability. (General Observation)

**iOS Support:**
The iOS version lacks guidance or a help section. Providing clear instructions or tutorials for iOS users would enhance accessibility. (Severity: 3)

***EndoLit***

**Outdated Design Philosophy:**
The app is designed like older social media platforms (e.g., Facebook, LinkedIn), but the overall design philosophy feels outdated, confusing, and inconsistent, which detracts from the user experience. (General Observation)

**Interactive Button Behavior:**
In the user profile menu, the "Connect" option disappears after clicking, and a modal dialog informs the user that the request has been sent. Consider replacing this with an interactive button that updates in real-time by changing its color and text (e.g., from "Connect" to "Requested") upon clicking. (Severity: 3)

**Broken Share Links:**
The "Share" option generates a link, but opening the link in a browser results in a "404 Not Found" error. Ensure the shared links are functional and direct to the intended content. (Severity: 3)

**Slow Menu Changes and Back Button Response:**
Switching between menus and pressing the back button is noticeably slow, often requiring multiple presses or waiting through a loading animation. This significantly impacts usability. Optimizing performance for these actions is necessary. (Severity: 3)

**User Profile Placement:**
Accessing the user’s profile is unintuitively placed under the “More” option at the bottom right. Relocating it to a clearly labeled “Profile” section would improve navigation. (Severity: 3)

**App Options Marker:**
Access to other app options is located in the top-right corner, but the marker is unclear and does not indicate its function effectively. Consider redesigning it for better visibility and recognition. (Severity: 2)

**Font Size Issue:**
The font size on the "About the App" page is too small, making it difficult to read. Adjusting the font size for better accessibility is recommended. (Severity: 2)

**Broken PDF Viewer:**
The "PDF Viewer" feature is frequently non-functional, with files failing to open or display correctly. Addressing this issue is critical to restoring functionality. (Severity: 4)

**Notification Repetition:**
Notifications appear twice each time, which can be distracting for users. Ensure that notifications are displayed only once per event. (Severity: 1)

***AAE Endocase***

**Small Font in "How to Use" Menu:**
The font size in the "How to Use" menu is too small, making it difficult to read for some users. Increasing the font size would improve readability and accessibility. (Severity: 1)

**Low-Quality Graphics:**
The graphics in the app are small and of low quality, which detracts from the overall user experience. Enhancing the resolution and size of graphics would make the app more visually appealing. (Severity: 2)

**Content Availability:**
The app provides some content within the app itself rather than merely redirecting users to a website. This is a positive feature and makes the app more functional and self-contained. (Severity: 1)

**Dental Endo Master**

**Video Playback Control:**The app requires students to close instructional videos every time they want to play them again. It would be better if the videos could be skipped after the first use, allowing for smoother navigation and usability. (Severity: 1)

**Endo10**

**Irrelevant Bottom Navigation Bar Visuals:**
The visuals on the bottom navigation bar do not accurately represent the sections they correspond to, leading to confusion during navigation. Updating the icons or visuals to better align with their respective sections is recommended. (Severity: 3)

**Inconsistent Signs and Symptoms Explanation:**
When signs and symptoms are inconsistent, the app simply displays "Inconsistent" without providing any explanation for the inconsistency. Including a detailed explanation or reasoning for the inconsistency would enhance the user experience and provide greater value. (Severity: 2)

***ADAT Endodontic Cram Cards***

**Progress Tracker Implementation:**Implement a progress tracker that shows users their completion status for each topic or section. This feature would help users monitor their learning progress and stay motivated. *(Severity: 2)*

**Navigation Enhancements:**Enhance navigation by adding features like a back button, the ability to bookmark flashcards, and options to review previously answered questions. These improvements would streamline user interaction and reduce friction. *(Severity: 2)*

**Modern Visual Design Update:**Update the visual design to a more modern and clean aesthetic, using consistent color schemes, readable fonts, and intuitive icons. This would enhance the overall user experience and visual coherence. *(Severity: 3)*

**User Feedback Feature Integration:**Incorporate a feature that allows users to report issues, suggest improvements, or ask questions directly within the app. This addition would foster user engagement and provide actionable insights for developers. *(Severity: 3)*

***ToothSOS***

**Overemphasis on “Refer a Friend” Link:**The "Refer a Friend" link on the landing page is too prominently displayed, despite being a relatively unimportant feature. Reducing its prominence would improve the focus on more critical app features. *(Severity: 2)*

**Inconsistent Titles:**The title card for "Injured Skin, Lips, and Gums" appears as "Lip and Soft Tissue Laceration," which is inconsistent and could confuse users. Aligning these titles would enhance clarity. *(Severity: 2)*

**Lack of Visual Differentiation:**The overall appearance and color schemes for the patient and professional pages are too similar, making it difficult for users to distinguish between them. Introducing distinct designs or colors for each page would reduce confusion. *(Severity: 3)*

**Redundant Version Switching:**

The option to switch between the patient and professional versions of the app is presented in three different ways, which is redundant and can clutter the interface. Streamlining these options into a single, intuitive method is recommended. *(Severity: 2)*

***Injured Tooth***

**Confusing Side Scrolling for Trauma Selection:**The side-scrolling design for selecting each trauma is confusing and unintuitive. A vertical, stationary layout would be more user-friendly and easier to navigate. *(Severity: 2)*

**Confusing Use of "Like" Button:**Using a "like" button as a graphic for descriptions is unclear and misleading. Consider replacing it with more appropriate and intuitive icons or visuals to better represent its function. (*Severity: 3)*

**Crowded and Inconsistent Bottom Navigation Icons:**The bottom navigation bar contains too many icons, and they vary in style, making the interface feel cluttered and visually inconsistent. Reducing the number of icons and standardizing their design would improve usability. *(Severity: 2)*

**Vague Information and Errors in Page Usage:**The "Using the App" page provides vague instructions, and users encounter errors when entering information. Additionally, some management options lead to unavailable web pages. Addressing these issues is critical to ensure reliability and functionality. *(Severity: 2)*

***AcciDent***

**Titles "Master Data" Emphasis:**The current titles for "Master Data" lack visual prominence. Enhancing their boldness and typographic emphasis would improve readability and user focus. *(Severity: 2)*

**Title "Calculator" Emphasis:**Similar to "Master Data," the "Calculator" title should be bolded and visually emphasized to ensure it stands out within the interface, aiding quick navigation. *(Severity: 2)*

**Tab Organization with Bounding Boxes/Dividers:**The current tab layout lacks clear visual separation. Implementing bounding boxes or dividers between tabs would enhance organizational clarity and user navigation. *(Severity: 2)*

**Subcategory-Specific Imagery:**Using distinct images tailored to each subcategory would improve user comprehension and make the interface more intuitive. *(Severity: 2)*
